# Intron retention coupled with nonsense-mediated decay is involved in cellulase biosynthesis in cellulolytic fungi

**DOI:** 10.1186/s13068-022-02141-x

**Published:** 2022-05-19

**Authors:** Yichen Gao, Ai-Ping Pang, Leyao Ma, Haiyan Wang, Samran Durrani, Bingzhi Li, Fu-Gen Wu, Fengming Lin

**Affiliations:** 1 State Key Laboratory of Bioelectronics, School of Biological Science and Medical Engineering, Southeast University, Nanjing, China; 2 Key Laboratory of Systems Bioengineering (Ministry of Education), School of Chemical Engineering and Technology, Tianjin University, Tianjin, China

**Keywords:** Lignocellulose, Cellulase, Alternative splicing, Retained intron, NMD

## Abstract

**Background:**

Knowledge on regulatory networks associated with cellulase biosynthesis is prerequisite for exploitation of such regulatory systems in enhancing cellulase production with low cost. The biological functions of intron retention (IR) and nonsense-mediated mRNA decay (NMD) in filamentous fungi is lack of study, let alone their roles in cellulase biosynthesis.

**Results:**

We found that major cellulase genes (*cel7a*, *cel7b*, and *cel3a*) exhibited concomitant decrease in IR rates and increase in their gene expression in *T*. *reesei* under cellulase-producing condition (cellulose and lactose) that was accompanied with a more active NMD pathway, as compared to cellulase non-producing condition (glucose). In the presence of the NMD pathway inhibitor that successfully repressed the NMD pathway, the mRNA levels of cellulase genes were sharply down-regulated, but the rates of IR in these genes were significantly up-regulated. Consistently, the cellulase activities were severely inhibited. In addition, the NMD pathway inhibitor caused the downregulated mRNA levels of two important genes of the target of rapamycin (TOR) pathway, *trfkbp12* and *trTOR1*. The absence of gene *trfkbp12* made the cellulase production in *T*. *reesei* more sensitive to the NMD pathway inhibitor.

**Conclusions:**

All these findings suggest that the IR of cellulase genes regulates their own gene expression by coupling with the NMD pathway, which might involve the TOR pathway. Our results provide better understanding on intron retention, the NMD pathway, and cellulase production mechanism in filamentous fungi.

**Supplementary Information:**

The online version contains supplementary material available at 10.1186/s13068-022-02141-x.

## Introduction

In multicellular organisms, a pre-mRNA is composed of exons intervened with introns. The interspersed introns can be removed through a large multiprotein splicing complex (the spliceosome) to yield the mature mRNA. During this splicing processing, a pre-mRNA can generate one or more functional transcripts by the alternative splicing (AS) process [1]. AS is a well-conserved mechanism for producing multiple isoforms of mRNA and/or proteins to increase the diversity of the transcriptome and proteome under varied physiological circumstances, therefore, overcoming the limitations caused by a finite genome and aiding an organism in accommodating to the changing environment [2]. There are seven main types of AS events: mutually exclusive, intron retention, cassette exons, alternative initiation, alternative termination, alternative donors, and alternative acceptors [3].

In intron retention (IR), introns that are supposed to be spliced appear in mature mRNA and subsequently participate in the translation process. At first, IR had been considered useless resulting from the malfunctioning of the spliceosome, and has been relatively ignored. Nevertheless, the role of IR has been studied more extensively in recent years. Retained introns affect the localization, translatability, stability, and function of the transcripts containing them [4]. IR plays an essential role in the regulations of gene expression, mRNA localization, tissue-specific protein diversity, alternative splicing, and dosage compensation of the X chromosome, participating in biological events, such as stress response, development, tissue differentiation, and disease [5, 6]. An increasing research has demonstrated that intron retention is widely found in animals, plants and fungi, serving as one of the effective strategies for post transcriptional regulation in eukaryotes [5]. In fungi, IR is reported to be involved in fungal cell complexity, pathogenicity [7], heat shock response [8], the nutrient sensing, such as glucose [9], and nitrogen source [10]. However, the function of IR in fungi has not been well studied, as compared to the extensive study of IR in animals and plants.

The intron-containing transcripts usually accommodate one or more premature termination codons (PTCs), which allow them to be recognized and degraded by nonsense-mediated mRNA decay (NMD), inhibiting the production of potentially harmful proteins [11]. The coupling of IR with NMD (IR-NMD) poses an additional post-transcriptional regulatory layer that can control mRNA quality and gene expression level [5]. IR-NMD can regulate a gene function via upregulating the expression of a non-functional NMD-targeted isoform of the gene, and consequently reducing the translation of the protein [12]. IR-NMD is rarely studied in filamentous fungi.

Cellulose is found widely in nature (leaves, grass, and wood) and waste materials (municipal wastes and agricultural wastes). The cellulase-mediated bioconversion of cellulose to fermentable sugars for biomass-derived biorefinery is potent, sustainable, and environment-friendly. Cellulase is a mixture of extracellular enzymes acting collaboratively for cellulose decomposition, majorly including endoglucanase (CMC; EC 3.2.1.4) cleaving cellulose in an endo-acting way and exhibiting a great affinity towards the soluble cellulose derivatives, cellobiohydrolase (CBH; EC 3.2.1.91) working as exoenzymes to generate cellobiose from cellulose, and β-glucosidase (BGL; EC 3.2.1.21) converting cellobiose to glucose [13, 14]. Cellulose is the efficient natural inducer for cellulase production by filamentous fungi, such as *Trichoderma reesei*, *Neurospora crassa*, *Aspergillus nidulans*, and *Penicillium decumbens*, followed by lactose, while glucose is the repressor of cellulase production. The knowledge on the regulatory molecular mechanism of cellulase synthesis in filamentous fungi is prerequisite for rationally engineering fungal strains to improve the production of cellulase and other proteins, such as pharmaceutical proteins and industrial enzymes, which has received increasing attention [15]. It has been reported that cellulase production is regulated by varied signal pathways, such as carbon catabolite repression (CCR) [16], calcium signal transduction pathway [17], and the TOR pathway [18]. When glucose exists, CCR is induced through the transcription factor CRE1 to almost completely block the cellulase production [16]. In contrast, the Ca^2+^ burst through calcium signal transduction pathway can strengthen the cellulase production and cell metabolism [17]. However, whether IR and NMD is involved in cellulase biosynthesis in filamentous fungi and how, remain totally unknown.

In this study, to investigate the function of IR-NMD in cellulase production in filamentous fungi, the mRNA levels and IR rates of the three major cellulase genes (*cel7a*, *cel7b*, and *cel3a*) in *T*. *reesei*, a well-known work horse for industrial cellulase production [19], were investigated under cellulase non-producing conditions together with the mRNA levels of the NMD pathway. A lower IR level with a more active NMD pathway was observed under cellulase-producing than under cellulase non-producing, suggesting a key role of IR-NMD in cellulase biosynthesis. To further verify this finding, the NMD pathway inhibitor was explored to repress the NMD pathway, leading to the increased IR rates and decreased mRNA levels of cellulase genes as well as significantly decreased cellulase production on cellulose. Moreover, the effect of the NMD pathway inhibitor on the phenotype of *T*. *reesei* and the TOR pathway was investigated. This study provides new knowledge on the regulation mechanism of cellulase production in terms of IR-NMD.

## Materials and methods

### Strains and culture condition

*T*. *reesei* RUT-C30 (CICC 13,052) was purchased from China Center of Industrial Culture Collection (CICC). *T*. *reesei* RUT-C30 activated with Sabouraud Dextrose Broth (SDB) for 48 h was cultivated in *Trichoderma* minimal medium (TMM) [20] containing NMD inhibitor for 7 days. The TMM medium was prepared as followed (all concentrations in g/L unless otherwise noted): ammonium sulfate, 4; potassium dihydrogen phosphate, 6.5; tween-80, 0.0186% (v/v); yeast extract, 0.25; tryptone, 0.75; maleic acid, 11.6; cellulose, 2% (w/t); zinc sulfate heptahydrate, 0.0014; manganese sulfate monohydrate, 0.0016; cobalt chloride hexahydrate, 0.002; ferrous sulfate heptahydrate, 0.005; urea, 1.00; calcium chloride, 0.60; magnesium sulfate, 0.60. The pH of TMM was adjusted to 5.8 ~ 6.0 by sodium hydroxide. The NMD pathway inhibitor caffeine was ordered from APExBIO. Actinomycin D was purchased from National Standard Material Center. All chemicals utilized in this research were ordered from Sigma-Aldrich, USA.

### Shake flask cultivation

*T*. *reesei* was cultured on potato dextrose agar (PDA) plate at 28 °C for 7 days to reach 10^7^/mL conidia. The conidia of 0.5 mL were inoculated into 10 mL SDB and incubated for 2 days at 28 oC with 220 rpm. Then 5 mL of the preculture were transferred to a 250 mL Erlenmeyer flask containing 50 mL TMM media containing different concentrations of the NMD pathway inhibitor (caffeine) as indicated in the text together with actinomycin D for stabilizing the mRNA level [21], and cultivated at 28 °C with 220 rpm for 7 days. Samples were taken every 24 h and centrifuged at 14,000 g for 10 min at 4 °C to separate the mycelia and the supernatant. The obtained mycelia were utilized for RNA extraction, DNA extraction and confocal observation, while the supernatants were used for cellulase activity assay and conidia counting. Strain RUT-C30 cultured in TMM with different carbon sources (cellulose, lactose and glucose) without the NMD pathway inhibitor was set as the controls.

### RNA extraction and real-time quantitative PCR

Total RNA of *T*. *reesei* RUT-C30 and caffeine-treated RUT-C30 were extracted using a fungal RNA extraction kit (Omega Bio-tek, Germany). HiScript® III RT SuperMix for qPCR (+ gDNA wiper) Kit (Vazyme, China) was used to obtain the first-strand cDNA. ChamQ Universal SYBR qPCR Master Mix Kit (Vazyme, China) was used to detect the relative RNA level by the ABI Stepone instrument Plus (ABI, Germany) with software Version 2.3 (ABI, Germany). The primers for qPCR were displayed in Additional file 1: Table S1. The gene *scar1* was selected as the reference gene for data normalization. The intron retention rate presented in percentage was calculated as follow: the mRNA level of the retained intron in the target gene was divided by the total mRNA level of the target gene.

### Enzyme assay and detection of DNA content

For the measurement and calculation of (hemi)cellulase activities, the spores were first pre-cultured in SDB broth at the content of 10^7^/mL for 48 h and then inoculated into TMM medium at the concentration of 10% for 7 days, the samples were drawn based on different requirements and then centrifuged at 14,000 g, 4 ℃, 10 min. The (hemi)cellulase activities of the supernatants were performed as described in our previous research [22] and the mycelia were used for DNA content detection.

### Microscopy observation

Hyphae of *T*. *reesei* RUT-C30 treated with NMD inhibitor at different time periods were loaded on a glass slide, covered with a cover glass, and observed on an inverted confocal laser scanning microscope SP8 (Leica, Germany) with a 100 X 1.4 NA oil-immersion objective. 405 nm was used as the excitation wavelength of light and the emission was detected in the range of 415–495 nm.

## Results

### Concomitant increase in IR rates and decrease in gene expression was observed for cellulase genes

**Fig. 1:**
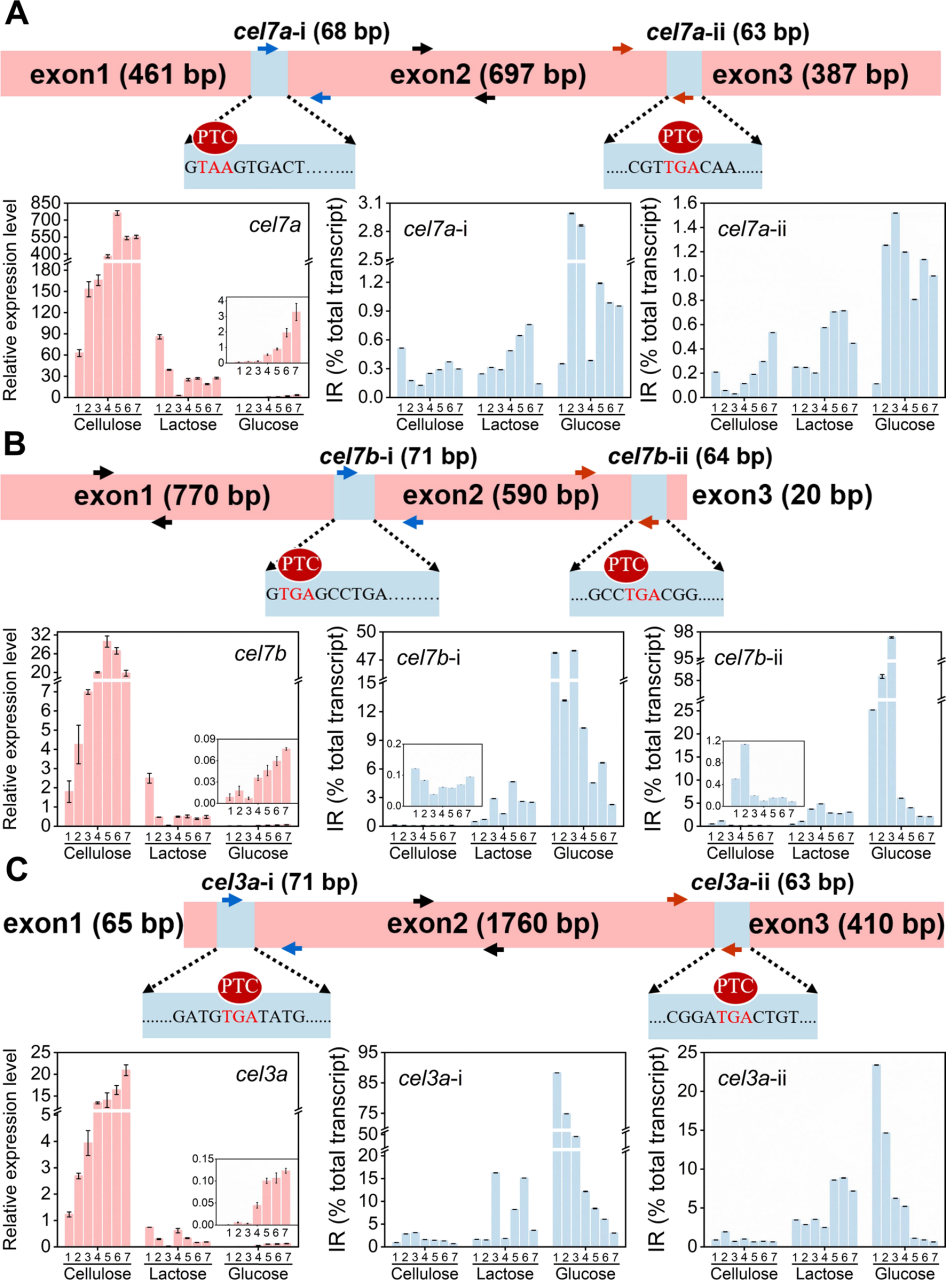
Relative expression and their retention rates of genes *cel7a*, *cel7b*, and *cel3a* in *T*. *reesei* RUT-C30 cultivated for 7 days in TMM containing 2% cellulose, 2% lactose, or 2% glucose. The primers for qPCR were marked in three colors (the red one: “the primers for the first intron”; the blue one: “the primers for the second intron”; the dark one: “the primers for the whole gene”). Data are represented as the mean of three independent experiments and error bars express the standard

To investigate the possible role of intron retention on cellulase production, we detected the dynamic mRNA levels and IR rates of cellulase genes under cellulase-producing conditions (cellulose and lactose) and cellulase non-producing condition (glucose) (Figs. 1 and Additional file 1: Fig. S1), including exoglucanase 1 (*cel7a*, CBH), endoglucanase I (*cel7b*, CMC), and β-glucosidase (*cel3a*, BGL). There are 2 introns in *cel7a* (*cel7a*-i and *cel7a*-ii), *cel7b* (*cel7b*-i and *cel7b*-ii), and *cel3a* (*cel3a*-i and *cel3a*-ii), respectively (Fig. 1). The total mRNA levels of *cel7a*, *cel7b*, and *cel3a* were the highest in *T*. *reesei* grown on cellulose, followed by that on lactose and glucose, respectively (Fig. 1A–C), which is consistent with the cellulase-inducing ability of cellulose, lactose, and glucose in the decreasing order. In contrast, the retention rates of all introns in *cel7a*, *cel7b* or *cel3a* were the lowest in *T*. *reesei* grown on cellulose, followed by that on lactose and glucose, respectively. On cellulose, where cellulase were necessary for the conversion of cellulose to glucose for cell growth, *cel7a*, *cel7b* or *cel3a* were highly expressed and their IR rates were all pretty low with a range from 0.03% to 3.12%. On lactose, a weaker cellulase inducer than cellulose, the retention rates of the two introns in *cel7a* (*cel7a*-i and *cel7a*-ii) were not changed in comparison with that on cellulose, while the retention rates of introns in *cel7b* and *cel3a* were increased moderately. Meanwhile, the mRNA levels of three cellulase genes were reduced in comparison with that on cellulose. On glucose that almost abolished cellulase production, the transcriptional levels of *cel7a*, *cel7b* or *cel3a* were markedly reduced, while the retention rates of introns in *cel7a*, *cel7b* or *cel3a* were all upregulated significantly, as compared to that on cellulose. The retention rates for *cel3a*-i and *cel3a*-ii were the maximum on day 1 in *T*. *reesei* grown on glucose, which was 88.3% and 23.4%, respectively. Then, the retention rates for *cel3a*-i and *cel3a*-ii were decreasing as glucose was consumed. The highest retention rates of *ce7b*-i and *cel7b*-ii on glucose were 48.0% and 97.4% on day 3, respectively. The retention rates of *cel7a*-i and *cel7a*-ii were all very low at all tested carbon sources in the range of 0.11–2.99%, indicating that the retention of the two introns in *cel7a* was not significant. It seems the IR rates of cellulase genes was negatively correlated with their gene expression, being much higher under cellulase non-producing condition than cellulase producing conditions. Obviously, intron retention of cellulase genes plays a crucial role in modulating cellulase biosynthesis.

### The NMD pathway is more active under cellulase-producing condition than cellulase non-producing condition

**Fig. 2:**
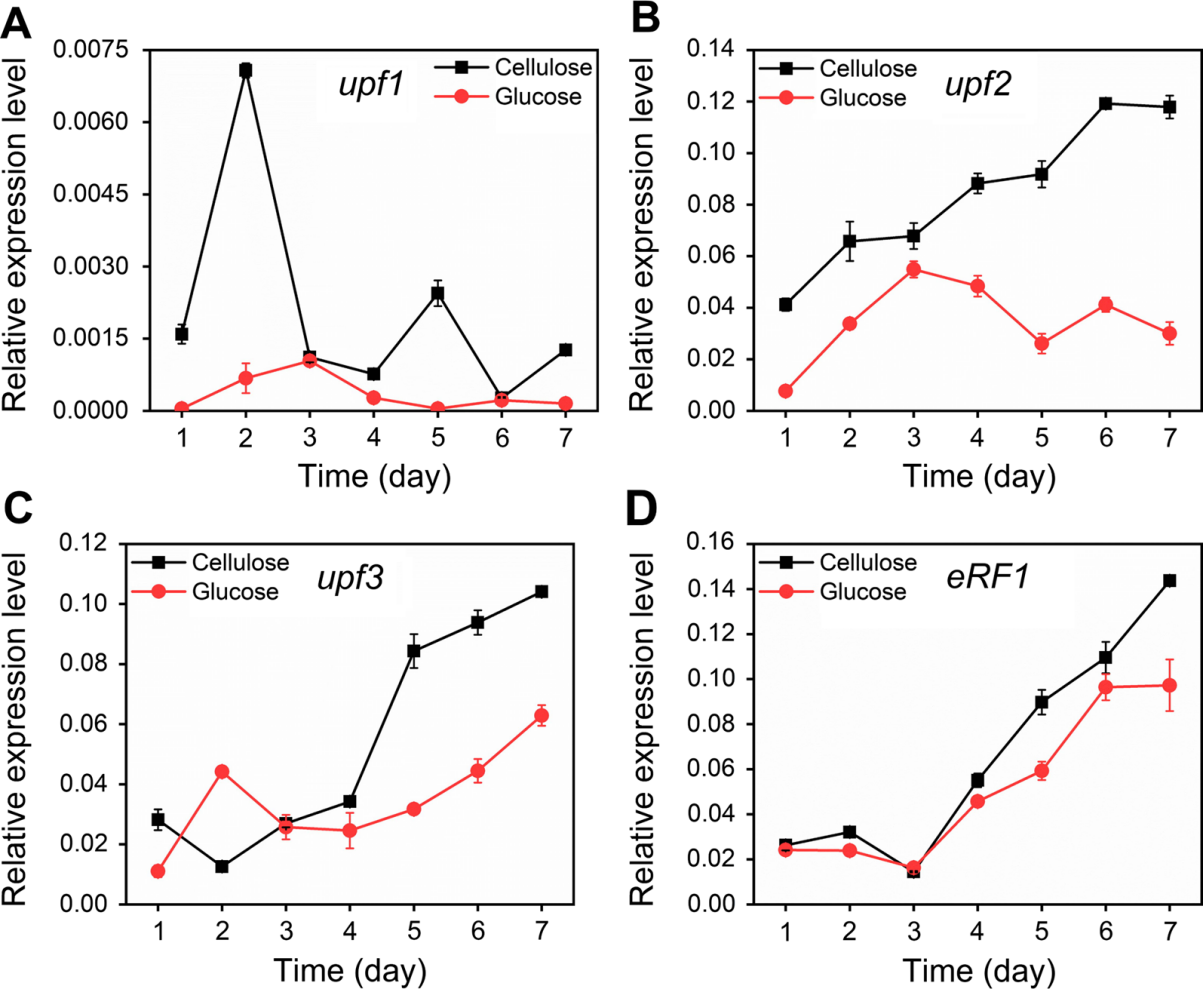
Relative expression levels of genes related to the NMD pathway in *T*. *reesei* RUT-C30 cultivated in TMM containing 2% cellulose or 2% glucose on days 1–7. Data are represented as the mean of three independent experiments and error bars express the standard

As the retained introns in cellulase genes *cel7a*, *cel7b*, and *cel3a* contain PTCs (Fig. 1), the intron-retaining transcripts of *cel7a*, *cel7b*, and *cel3a* might be the targets of the NMD pathway. In the NMD pathway, proteins UPF1, UPF2, and UPF3 constitute the core NMD complex with UPF1 as the core factor [23]. Eukaryotic release factor 1 and 3 (ERF1 and ERF3) can bind to UPF1 to induce the degradation of mRNA [24]. We detected the mRNA transcripts of gene *upf1*, *upf2*, *upf3*, *eRF1* and *eRF3* in RUT-C30 cultured on TMM with 2% cellulose (cellulase-producing condition) or 2% glucose (cellulase non-producing condition) by RT-qPCR (Fig. 2). The mRNA expression of *eRF3* was not detected under all the tested conditions. Most of the time, the relative expression level of gene *upf1*, *upf2*, *upf3*, and *eRF1* on 2% cellulose was higher than its corresponding mRNA levels on 2% glucose on the same day (Fig. 2). However, the expression levels of *upf1* were much lower than that of the other three genes *upf2*, *upf3*, and *eRF1* under different carbon sources. Except *upf1* that displayed fluctuated expression levels, the other three genes all showed upward trends through the whole fermentation on cellulose. On glucose, the transcriptional levels of *upf1* and *upf2* were upregulated in the first 3 days during which glucose was consumed completely, and decreased in next 4 days, while *upf3* and *eRF1* exhibited increasing trends similar to that on cellulose. These results indicated that the NMD pathway was more activated under cellulase-producing condition than cellulase non-producing condition.

### The intron retention rates of cellulase genes were increased when the NMD pathway was repressed by its inhibitor

**Fig. 3:**
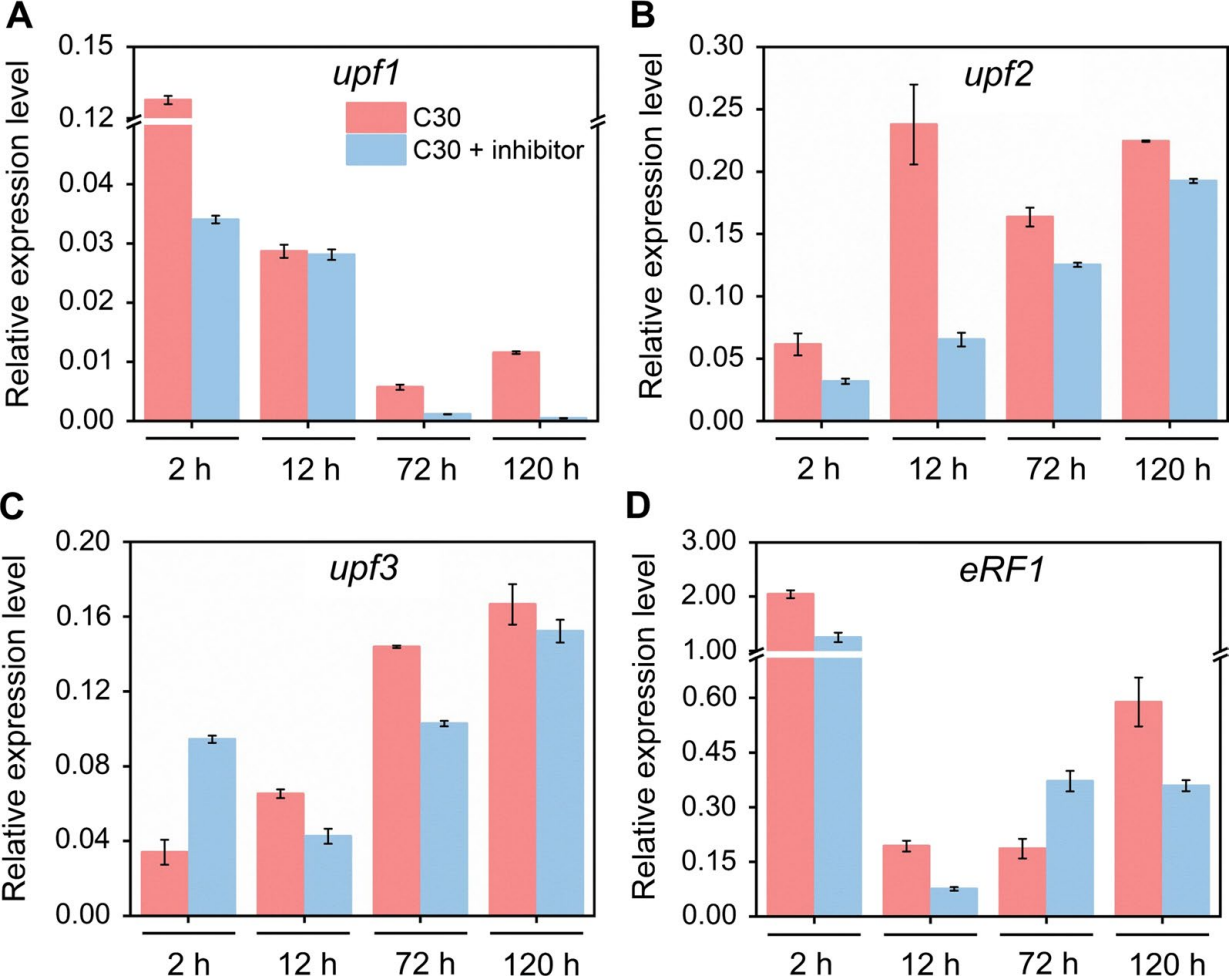
Relative expression of genes in the NMD pathway in *T*. *reesei* RUT-C30 cultivated for 2 h, 12 h, 72 h, and 120 h in TMM containing 2% cellulose with and without NMD pathway inhibitor (5 mM caffeine). Data are represented as the mean of three independent experiments and error bars express the standard

Next, we measured the effect of the NMD pathway inhibitor (caffeine) on the mRNA levels of genes *upf1*, *upf2*, *upf3*, and *eRF1* at different fermentation times (Fig. 3). The mRNA levels of *upf1* were significantly decreased at 2, 72, and 120 h, but was not changed at 12 h. The transcription levels of *upf2* were reduced noticeably at all tested timepoints. The expression of *upf3* was upregulated at 2 h, downregulated at 12 and 72 h, and then unchanged at 120 h. The mRNA abundance of *eRF1* were decreased remarkably at 2, 12, and 120 h, but increased at 72 h. These results implied that the active NMD pathway under cellulase-producing condition was probably inhibited in *T*. *reesei* RUT-C30 treated with the NMD inhibitor.

**Fig. 4:**
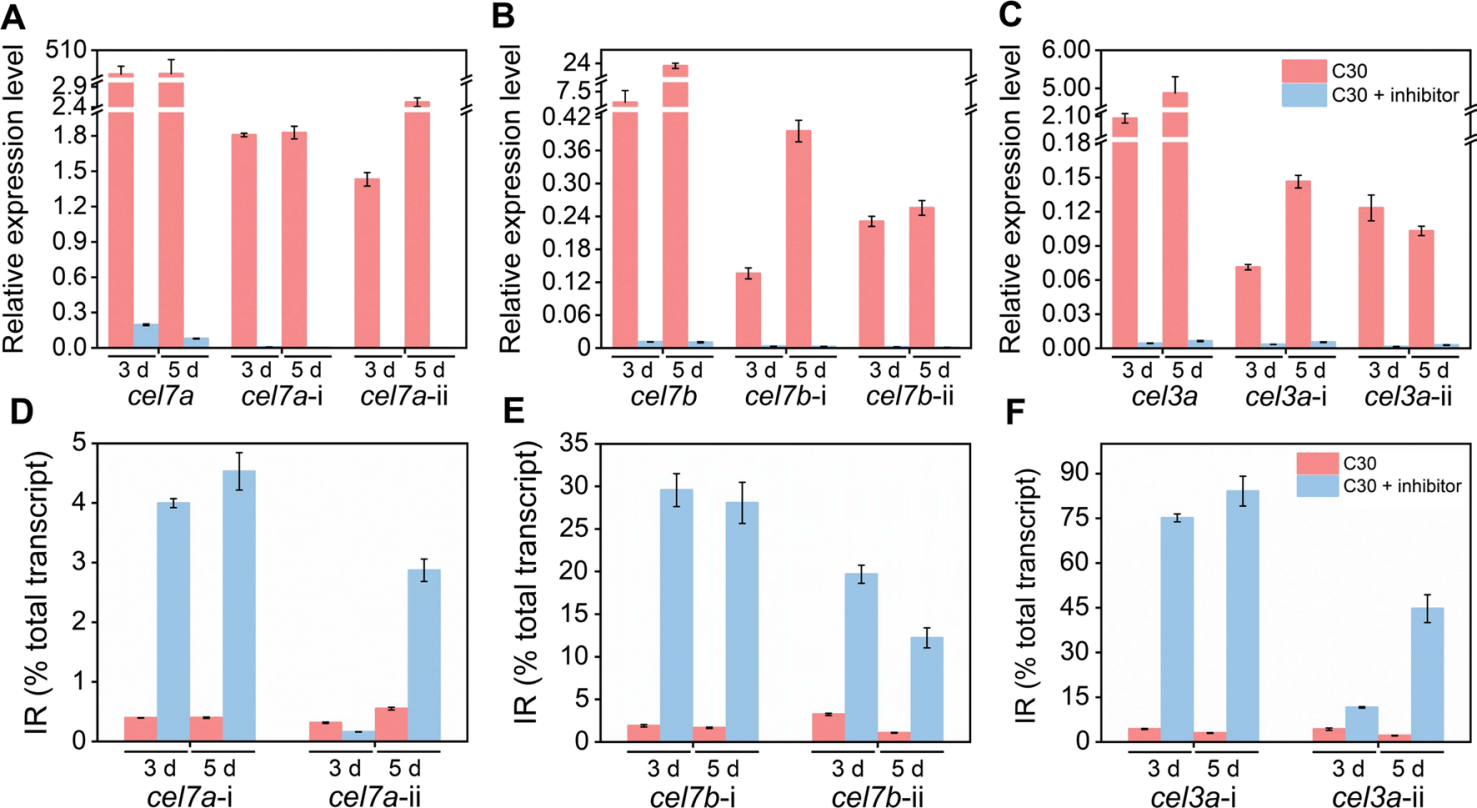
Relative expression levels and IR rates of cellulase genes in *T*. *reesei* RUT-C30 cultivated for 3 days and 5 days in TMM + 2% cellulose with and without the NMD pathway inhibitor (5 mM caffeine). Data are represented as the mean of three independent experiments and error bars express the standard

Given that the function of NMD is to degrade mRNA containing PTCs which might be caused by IR during the alternative splice process, the repression of the NMD pathway might influence the IR degrees of the major cellulase genes and their mRNA levels. Thus, we detected the mRNA levels and IR rates of *cel7a*, *cel7b*, and *cel3a* in *T*. *reesei* grown on cellulose in the presence of the NMD pathway inhibitor (Fig. 4). The transcription levels of *cel7a*, *cel7b*, and *cel3a* were sharply decreased on day 3 and day 5 in RUT-C30 treated with the NMD pathway inhibitor (Fig. 4A–C). On the contrary, on day 3 after the treatment of the NMD inhibitor, all introns of *cel7a*, *cel7b*, and *cel3a* were increased sharply in RUT-C30 treated with the NMD pathway inhibitor in comparison with that in untreated RUT-C30, except that the retention rate of *cel7a*-ii was decreased (Fig. 4D–F). The retention rates of *cel7a*-i, *cel7a*-ii, *cel7b*-i, *cel7b*-ii, *cel3a*-i, and *cel3a*-ii were increased from 0.39% to 4.00%, from 0.31% to 0.16%, from 1.90% to 29.56%, from 3.23% to 19.68%, from 3.48% to 78.91%, from 6.03% to 34.19%, respectively. Similarly, the significantly increased retention rates of all the introns in *cel7a*, *cel7b*, and *cel3a* were observed on day 5 in *T*. *reesei* with the treatment of the NMD pathway inhibitor. Interestingly, the retention rate of *cel3a*-i was much higher than that of *cel3a*-ii, which suggested that *cel3a*-i was easier to retain in mRNA than *cel3a*-ii in the presence of the NMD pathway inhibitor (Fig. 4F). In *T*. *reesei* treated with the NMD pathway inhibitor on day 5, the highest retention rate belonged to *cel3a*-i, followed by *cel3a*-ii, *cel7b*-i, *cel7b*-ii, *cel7a*-i, and *cel7a*-ii in a decreasing order. Clearly, the IR rates of cellulase genes *cel7a*, *cel7b*, and *cel3a* were markedly upregulated by the NMD pathway inhibitor, while the mRNA levels of these genes were decreased noticeably.

### The repression of the NMD pathway led to the notable inhibition of cellulase production

**Fig. 5:**
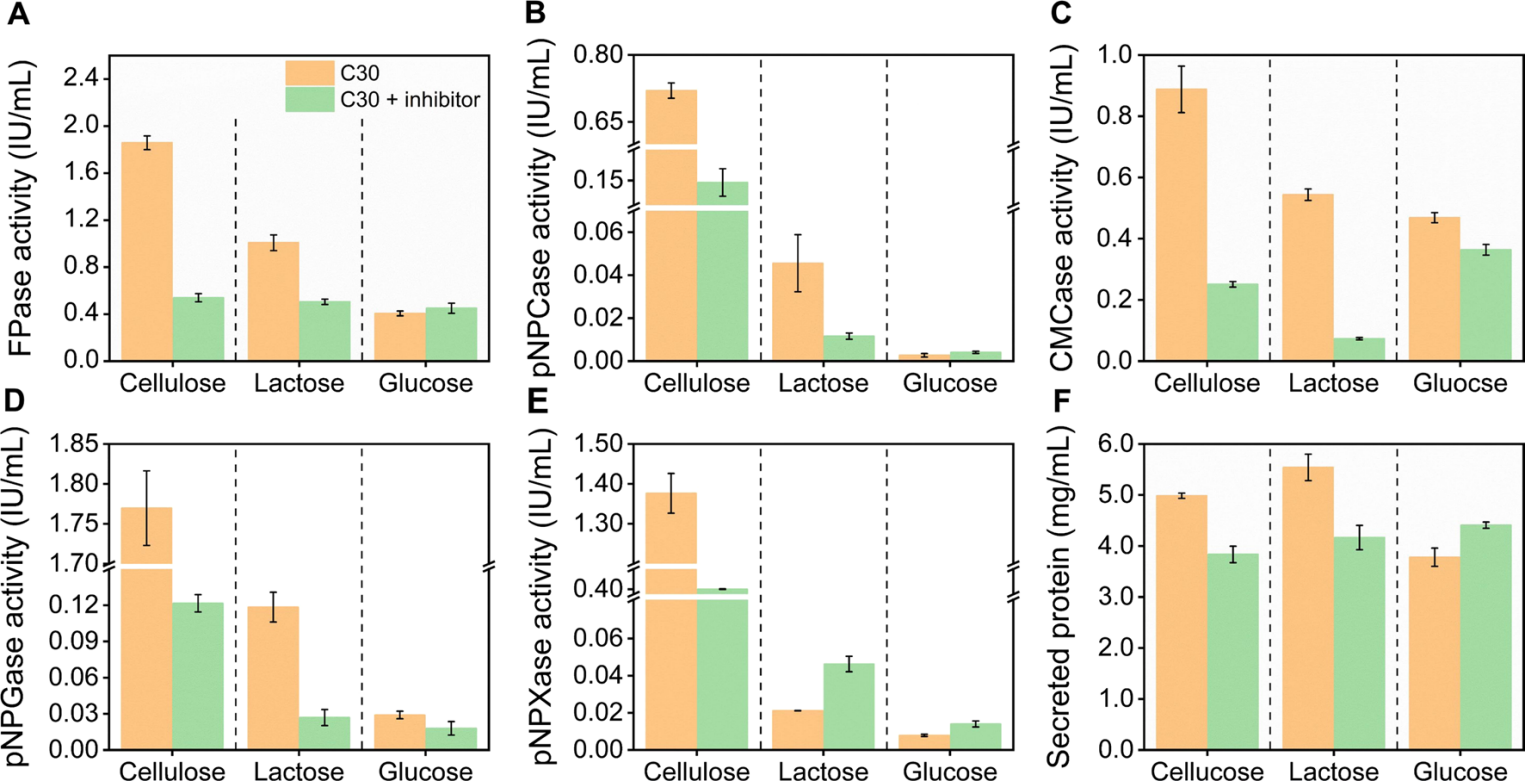
Cellulase activities of *T*. *reesei* RUT-C30 grown on different carbon sources with and without the NMD pathway inhibitor (5 mM caffeine), including **A** FPase; **B** pNPCase; **C** CMCase; **D** pNPGase; **E** pNPXase; and **F** secreted protein. The samples were taken on day 5 when 2% cellulose was utilized as the carbon source, and on day 3 when 2% lactose/glucose as the carbon source. Data are represented as the mean of three independent experiments and error bars express the standard

Inspired by the dramatic decrease of the mRNA levels of the major cellulase genes in the presence of the NMD pathway inhibitor, we speculated that the cellulase production would also be reduced. With this in mind, the cellulase activities of *T*. *reesei* RUT-C30 cultured on different carbon sources were measured in the presence of the NMD pathway inhibitor (Fig. 5). The (hemi)cellulase activities including FPase activity (the filter paper activity), pNPCase activity (the CBH activity), CMCase activity (the CMC activity), pNPGase activity (the β-glucosidase activity), pNPXase activity (the β-xylosidase activity), and the secreted protein concentration were all sharply declined in *T*. *reesei* RUT-C30 cultured on cellulose with the NMD pathway inhibitor on day 5 (Fig. 5). A similar inhibition effect of the NMD pathway inhibitor on cellulase activities was observed in *T*. *reesei* cultured on lactose for day 3 (Fig. 5). However, the pNPXase activity on lactose was increased after the addition of the NMD pathway inhibitor (Fig. 5E). When using glucose as the sole carbon source, the (hemi)cellulase activities were not affected significantly in the presence of the NMD pathway inhibitor (Fig. 5). Collectively, the NMD pathway inhibitor inhibited the (hemi)cellulase production in *T*. *reesei* RUT-C30 cultivated on cellulose or lactose, indicating the NMD pathway plays an important role in the production of (hemi)cellulase. This data was consistent with the notable reduction of cellulase genes at the mRNA level upon the addition of the NMD pathway inhibitor (Fig. 4).

### Phenotype profiling of *T*. *reesei* RUT-C30 with the treatment of the NMD pathway inhibitor

**Fig. 6:**
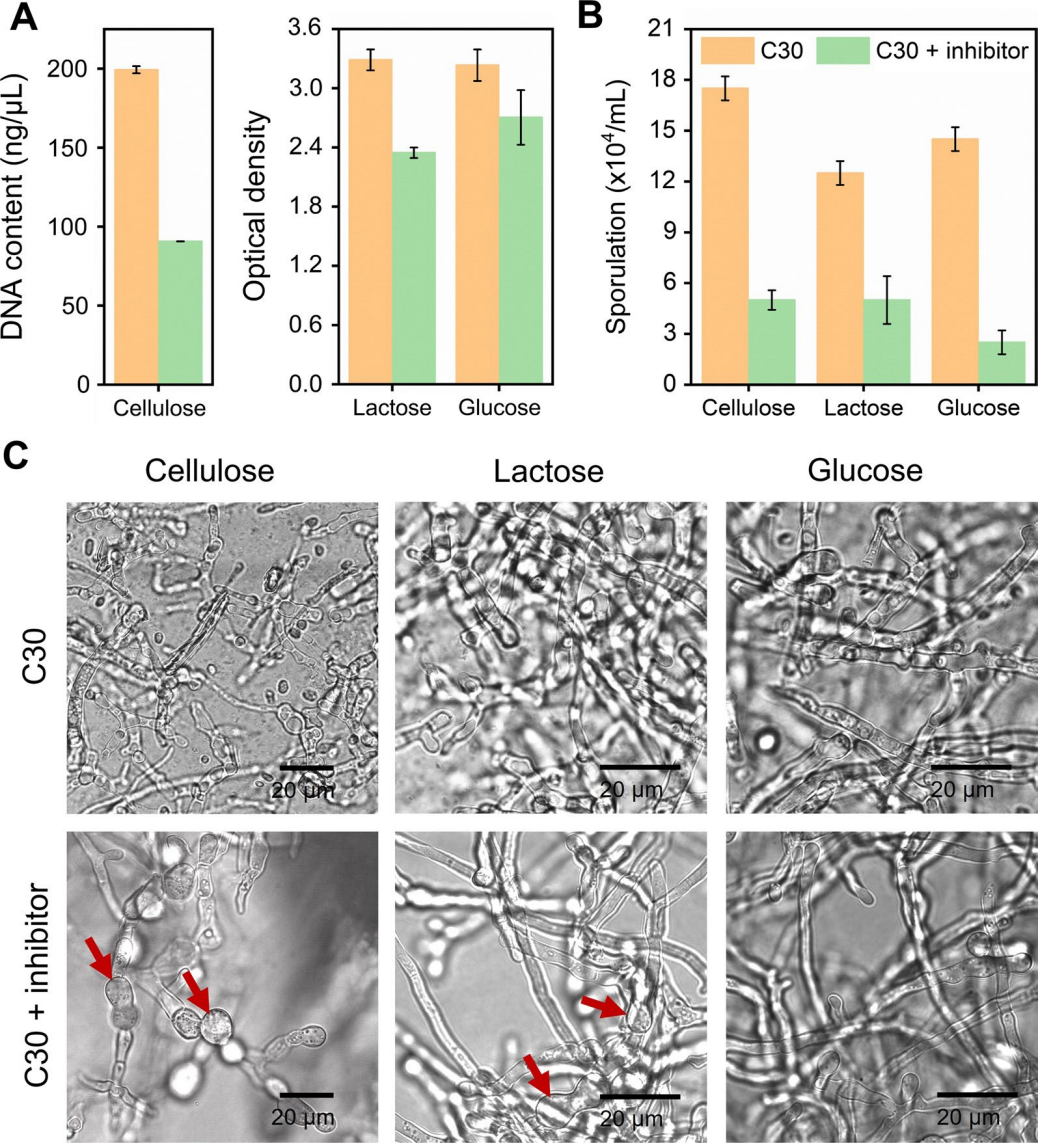
Phenotype of *T*. *reesei* RUT-C30 cultivated in TMM containing 2% cellulose, 2% lactose, and 2% glucose, respectively, in the presence of the NMD pathway inhibitor, including **A** mycelial growth; **B** sporulation; and **C** mycelial morphology. Red arrows pointed to the swollen mycelia after the NMD inhibitor treatment. All the samples were taken on day 5 under cellulose condition and on day 3 under lactose/glucose condition. Data are represented as the mean of three independent experiments and error bars express the standard

We investigated the impact of the NMD pathway inhibitor on the cell growth, sporulation, and morphology of *T*. *reesei* grown on different carbon sources (Fig. 6). Regardless the carbon sources, the cell growth of *T*. *reesei* were obviously decreased in the presence of the NMD pathway inhibitor (Fig. 6A). Similarly, the number of spores in the supernatants of RUT-C30 treated with the NMD inhibitor were sharply decreased (Fig. 6B). After the addition of the NMD pathway inhibitor, the spore amount of *T*. *reesei* proliferated on cellulose, lactose, and glucose were notably decreased, only 28.6%, 40%, and 17.2% of that of the untreated *T*. *reesei* (17.5 × 10^4^/mL, 12.5 × 10^4^/mL, and 14.5 × 10^4^/mL). Moreover, the confocal laser scanning microscope (CLSM) was used to investigate the effect of the NMD pathway inhibitor on the mycelial morphology of *T*. *reesei* RUT-C30 grown on cellulose, lactose and glucose (Fig. 6C). No matter what the carbon source was, the mycelia of the untreated *T*. *reesei* RUT-C30 were grown well and distributed evenly, whose shape was mostly slender, while the morphology of mycelia became swollen with shorter mycelia length on 2% cellulose or lactose in the presence of the NMD inhibitor. However, this phenomenon was not observed in RUT-C30 cultured on glucose in the presence of the NMD pathway inhibitor, implying that the morphology of *T*. *reesei* RUT-C30 cultured on cellulose was more sensitive to the NMD pathway inhibitor than on lactose or glucose. Collectively, the growth and sporulation ability of *T*. *reesei* were significantly suppressed by the NMD pathway inhibitor together with altered morphology.

### The TOR pathway was influenced by the NMD pathway inhibitor

**Fig. 7:**
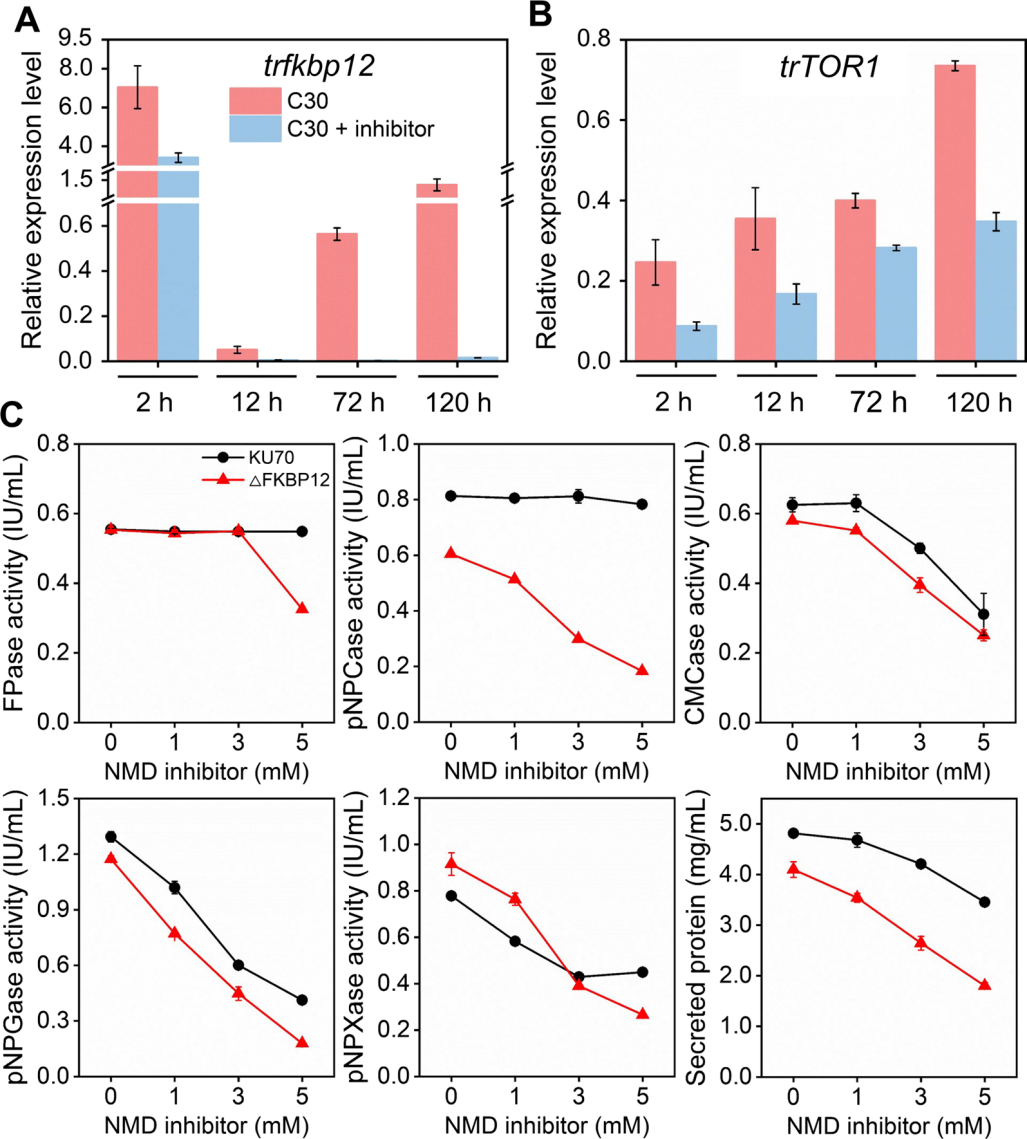
Relative expression of genes **A** *trfkbp12* and **B** *trTOR1* in the TOR pathway in *T*. *reesei* RUT-C30 cultivated at 2 h, 12 h, 72 h, and 120 h in TMM containing 2% cellulose with and without the NMD pathway inhibitor (5 mM caffeine). **C** Cellulase activities of strains KU70 and △*trfkbp12* treated with the NMD pathway inhibitor at different concentrations (1, 3, and 5 mM caffeine), respectively. Data are represented as the mean of three independent experiments and error bars express the standard

The mRNA levels of the two key genes in the TOR pathway (*trfkbp12* and *trTOR1*) in *T*. *reesei* grown on cellulose in the presence of the NMD pathway inhibitor were measured by RT-qPCR (Fig. 7A, B). The transcription levels of *trfkbp12* and *trTOR1* were markedly reduced by the NMD pathway inhibitor during the whole fermentation process. Furthermore, the *trfkbp12-*knockout strain △*trfkbp12* showed lower cellulase activities and protein secretion than the parental strain KU70 after the treatment of the NMD inhibitor (Fig. 7C), implying that the absence of gene *trfkbp12* makes *T*. *reesei* more sensitive to the NMD pathway inhibitor. Gene *trfkbp12* might play a role in antagonizing the cellulase-depression impact of the NMD pathway inhibitor.

## Discussion

**Fig. 8:**
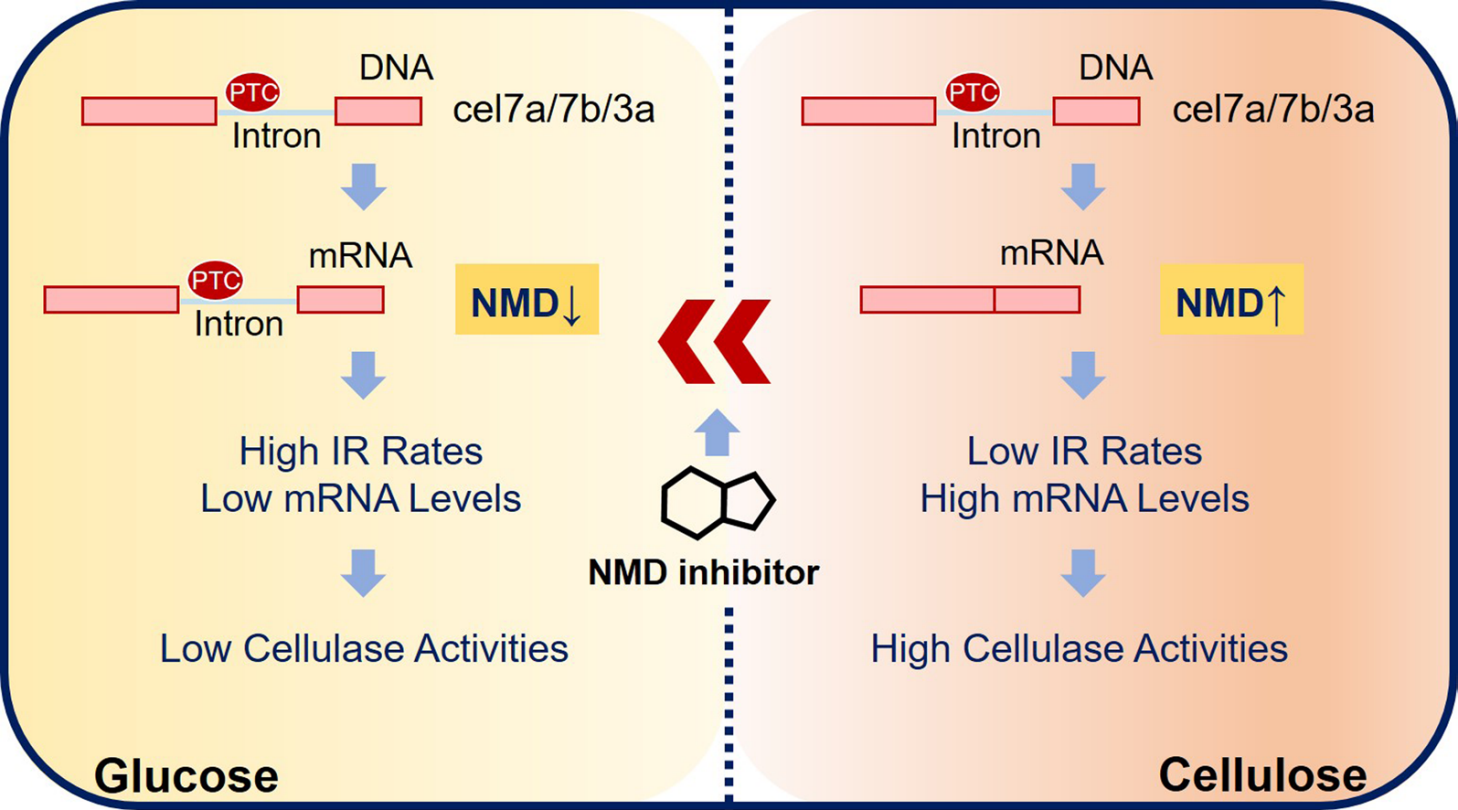
Role of IR-NMD RNA surveillance machinery in cellulase production in *T*. *reesei* RUT-C30 using cellulose

IR is the prevalent type of alternative splicing in fungi and plant, whereas exon skipping (ES) is the most common kind of alternative splicing in animals [7, 25–27]. Although IR is often shown to be involved in the regulation of vital developmental events in plants and animals [28, 29], the functional involvement of IR in lower eukaryotic filamentous fungi has not been well studied. For the first time, we investigated the role of IR and NMD in cellulase biosynthesis in filamentous fungus (Fig. 8).

IR is well known to play a fundamental role in the fine-tuning of gene expression. During the differentiation of embryonic stem cells into neural progenitors, IR facilitated the increased expression of genes with neuron-specific functions and the decreased expression of genes related with cell cycle progression [29]. In *C*. *neoformans*, IR represents an additional layer of gene expression regulation in response to environmental changes [30]. Particularly, the association of high IR rates with down-regulation of gene expression have been well reported [31]. The high IR rate of α-glucosidase reduced its production in *Aspergillus niger* [31]. The artificial elimination of the retained introns in gene *Pab2p* in *Cryptococcus neoformans* results in enhanced gene expression [32]. Retained introns in a murine neuroblastoma cell line was shown to negatively regulate genes with neural-associated functions [33]. Similarly, we observed a negative correlation between cellulase gene expression and their intron retention rates in *T*. *reesei* here (Fig. 8). The IR rates of *cel7a*, *cel7b*, and *cel3a* were decreased under cellulase-producing condition that has high expression of cellulase genes, as compared to cellulase non-producing condition with low expression of cellulase genes, implicating that IR is involved in coordinating cellulase gene expression.

In animals, IR functions widely to decrease the levels of transcripts that are not relevant for the physiology of the cell or tissue type [29]. An identical function of IR in the three major cellulase genes was found in this study. Cellulase genes *cel7a*, *cel7b*, and *cel3a* are required for *T*. *reesei* cultivated on cellulose to produce cellulase for the conversion of cellulose to glucose for cell growth and survival, where the expression of *cel7a*, *cel7b*, and *cel3a* were very high with low IR rates. Instead, when *T*. *reesei* was grown on glucose, genes *cel7a*, *cel7b*, and *cel3a* are not required and their mRNA abundance is low with high IR rates. Clearly, IR also functions to reduce the expression of relatively low abundance transcripts that lack physiological relevance to the *T*. *reesei* cells.

Intron retention is considered as a critical mechanism that independently reduces gene expression in normal biology. Increased IR is generally correlated with reduced mRNA and protein level. Increased IR observed under cellulase-repression condition might present an additional layer of gene expression regulation at post-transcriptional level to carbon catabolite repression at transcriptional level, ensuring the cellulase genes are not translated in *T. reesei* grown on glucose when cellulase is not required. In animals, IR functions widely to decrease the levels of transcripts that are not relevant for the physiology of the cell or tissue type [29]. Meanwhile, IR may control translation of cellulase genes in a cellulose specific manner, that is, by retaining introns in the mature mRNAs of cellulase genes and only splicing when necessary, to induce rapid protein production in *T. reesei* once encountering cellulose, which has been reported for genes related to development in plants and sea-anemones [4, 34].

IR events tune gene expression via different mechanisms. IR is associated with lower protein levels due to intron-retaining transcripts are either degraded by NMD or are not actively translated after escaping NMD [35]. In contrast, another set of IR events were reported to control gene expression by nuclear retention and exosome-mediated degradation of the intron-containing mRNA [33]. We found that the NMD pathway takes a great part in the control of the intron-containing cellulase genes. The active NMD pathway was found under cellulase condition when the cellulase genes were highly expressed with low IR rates. Moreover, in *T*. *reesei* grown on cellulose with the NMD pathway repressed by its inhibitor, the concomitantly increased IR rates and decreased cellulase gene expression was observed, which led to the notable inhibition of cellulase production. The IR of cellulase genes is coupled to the NMD pathway to regulate their own expressions when different carbon sources are utilized. It seems that the active NMD pathway with low intron retention rates of cellulase genes are essential for cellulase biosynthesis in filamentous fungus. This finding supports and expands the previously demonstrated function of IR coupled to NMD in regulating gene expression of arginine/serine rich splicing factor in animals [36], functionally relevant genes in granulocyte differentiation [37], and circadian rhythm-related genes in plants [38]. IR coupled to NMD can orchestrate gene expression, which is independent on transcriptional regulation. For instance, the coupling of IR to NMD has been reported to coordinate a number of functionally relevant genes in granulocyte differentiation, being required for normal granulopoiesis [37]. Meanwhile, tissues deficient in NMD exhibited a notable rising in low-abundant PTC-containing transcripts [39, 40]. In this study, we demonstrated that the expression of cellulase genes is regulated by IR-NMD RNA surveillance machinery (Fig. 8).

The TOR (Target of Rapamycin) signaling pathway is considered to be an important signaling hub coordinating the response of cell growth and metabolism to environmental factors, such as growth factors and nutrients in eukaryotic cells [41]. The peptidyl–prolyl cis/trans isomerase FKBP12 first binds to rapamycin to form a complex that subsequently interacts with the TOR kinases, such as TOR1, and inhibits their functions [42]. The expression levels of genes *trFKBP12* and *trTOR1* were significantly reduced in the presence of the NMD inhibitor. Knockout of gene *trFKBP12* increased the sensitivity of *T*. *reesei* to the NMD inhibitor, as indicated by the decreased cellulase production in strain ΔtrFKBP12 with the treatment of the NMD inhibitor, as compared to the parental strain KU70. Nevertheless, it is unknown how the TOR pathway is involved in the regulation of cellulase production by IR-NMD. The TOR growth-signalling network has been reported to be closely related to the regulation of growth by excised linear introns in yeast [43].

## Conclusions

In summary, we investigated the effect of IR of major cellulase genes *cel7a*, *cel7b*, and *cel3a* on their own gene expression and cellulase production in *T*. *reesei* RUT-C30*.* The much higher expression levels of these cellulase genes was concomitant with their lower IR rates under cellulase-producing condition (cellulose and lactose) than cellulase non-producing condition (glucose). It seems that the IR of cellulase genes acts to decrease the levels of cellulase transcription that are less or not required for the physiology of the *T*. *reesei* cells grown on glucose. Meanwhile, the NMD pathway was more active in *T*. *reesei* on cellulose than on glucose. When the NMD pathway was blocked by its inhibitor in *T*. *reesei* cultivated on cellulose, the transcriptional levels of cellulase genes were notably reduced and their IR rates were markedly increased, leading to the drastic drop of cellulase production. This further evidence that IR was linked with the NMD pathway to tune expression of cellulase genes at the post-transcriptional level. Meanwhile, the growth and sporulation ability of *T*. *reesei* cultured on cellulose were noticeably inhibited by the NMD pathway inhibitor together with altered morphology. Furthermore, the NMD pathway inhibitor reduced the mRNA levels of *trfkbp12* and *trTOR1*, two crucial genes in the TOR pathway*.* Meanwhile, the absence of gene *trfkbp12* compounded the inhibition effect of the NMD pathway inhibitor on the cellulase production using cellulose. All these results indicate that the IR of cellulase genes regulates their own gene expression by coupling with the NMD pathway to determine cellulase biosynthesis in *T*. *reesei*, which might be facilitated by the TOR pathway. These observations have implications for our understanding of IR-NMD-mediated RNA regulation of cellulase, and will help in the design of cellulase-hyperproducing strains in industry.

## Supplementary Information

**Additional file 1: Table S1**. Primers used for RT-qPCR in this study. **Fig. S1**. Relative levels of introns in *cel7a*, *cel7b*, and *cel3a* in *T*. *reesei* RUT-C30 cultivated for 7 days in TMM containing 2% cellulose, 2% lactose or 2% glucose. Data are represented as the mean of three independent experiments and error bars express the standard.

## Abbreviations

IR: Intron retention
NMD: Nonsense-mediated mRNA decay
AS: Alternative splicing
PTCs: Premature termination codons
IR-NMD: IR with NMD
CMC: Endoglucanase
CBH: Cellobiohydrolase
BGL: β-Glucosidase
CCR: Carbon catabolite repression
CICC: Center of industrial culture collection
SDB: Sabouraud dextrose broth
TMM: *Trichoderma* Minimal medium
PDA: Potato dextrose agar
FPase: The filter paper activity
pNPCase: The CBH activity
CMCase: The CMC activity
pNPGase: The β-glucosidase activity
pNPXase: The β-xylosidase activity
CLSM: Confocal laser scanning microscope
ES: Exon skipping
TOR: Target of rapamycin

## References

[1] Riolo G, Cantara S, Ricci C (2021). What’s wrong in a jump? Prediction and validation of splice site variants. Methods Protoc.

[2] Cherry S, Lynch KWJG (2020). Development: alternative splicing and cancer: insights, opportunities, and challenges from an expanding view of the transcriptome. Genes Dev.

[3] Ramani AK, Calarco JA, Pan Q, Mavandadi S, Wang Y, Nelson AC, Lee LJ, Morris Q, Blencowe BJ, Zhen M (2011). Genome-wide analysis of alternative splicing in Caenorhabditis elegans. Genome Res.

[4] Boothby TC, Zipper RS, van der Weele CM, Wolniak SM (2013). Removal of retained introns regulates translation in the rapidly developing gametophyte of Marsilea vestita. Dev Cell.

[5] Ge Y, Porse BT (2014). The functional consequences of intron retention: alternative splicing coupled to NMD as a regulator of gene expression. BioEssays.

[6] Buckley PT, Khaladkar M, Kim J, Eberwine J (2014). Cytoplasmic intron retention, function, splicing, and the sentinel RNA hypothesis. Wiley Interdiscip Rev RNA.

[7] Grutzmann K, Szafranski K, Pohl M, Voigt K, Petzold A, Schuster S (2014). Fungal alternative splicing is associated with multicellular complexity and virulence: a genome-wide multi-species study. DNA Res.

[8] Hossain MA, Rodriguez CM, Johnson TL (2011). Key features of the two-intron Saccharomyces cerevisiae gene SUS1 contribute to its alternative splicing. Nucleic Acids Res.

[9] Hossain MA, Claggett JM, Edwards SR, Shi A, Pennebaker SL, Cheng MY, Hasty J, Johnson TL (2016). Posttranscriptional regulation of Gcr1 expression and activity is crucial for metabolic adjustment in response to glucose availability. Mol Cell.

[10] Ishida K, Kuboshima M, Morita H, Maeda H, Okamoto A, Takeuchi M, Yamagata Y (2014). Diversity in mRNA expression of the serine-type carboxypeptidase ocpG in Aspergillus oryzae through intron retention. Biosci Biotechnol Biochem.

[11] Wong JJ, Au AY, Ritchie W, Rasko JE (2016). Intron retention in mRNA: No longer nonsense: known and putative roles of intron retention in normal and disease biology. BioEssays.

[12] Dai Y, Li W, An L (2016). NMD mechanism and the functions of Upf proteins in plant. Plant Cell Rep.

[13] Dos Santos AC, Ximenes E, Kim Y (2019). Ladisch MRJTib: Lignin–enzyme interactions in the hydrolysis of lignocellulosic biomass. Trends Biotechnol.

[14] Contreras F, Pramanik S, M Rozhkova A, N Zorov I, Korotkova O, P Sinitsyn A, Schwaneberg U, D Davari M (2020). Engineering robust cellulases for tailored lignocellulosic degradation cocktails. Int J Mol Sci.

[15] Gabriel R, Thieme N, Liu Q, Li F, Kohler LT, Harth S, Jecmenica M, Ramamurthy M, Gorman J, Simmons BA (2021). The F-box protein gene exo-1 is a target for reverse engineering enzyme hypersecretion in filamentous fungi. Proc Natl Acad Sci U S A.

[16] Portnoy T, Margeot A, Linke R, Atanasova L, Fekete E, Sándor E, Hartl L, Karaffa L, Druzhinina IS, Seiboth B (2011). The CRE1 carbon catabolite repressor of the fungus Trichoderma reesei: a master regulator of carbon assimilation. BMC Genom.

[17] Chen Y, Fan X, Zhao X, Shen Y, Xu X, Wei L, Wang W, Wei D (2021). cAMP activates calcium signalling via phospholipase C to regulate cellulase production in the filamentous fungus Trichoderma reesei. Biotechnol Biofuels.

[18] Pang AP, Wang H, Zhang F, Hu X, Wu FG, Zhou Z, Wang W, Lu Z, Lin F (2021). High-dose rapamycin exerts a temporary impact on T. reesei RUT-C30 through gene trFKBP12. Biotechnol Biofuels.

[19] Do Vale LHF, Filho EXF, Miller RNG, Ricart CAO, de Sousa MV: Cellulase systems in trichoderma. In: Biotechnology and biology of trichoderma. 2014. p. 229–244.

[20] Minty JJ, Singer ME, Scholz SA, Bae CH, Ahn JH, Foster CE, Liao JC, Lin XN (2013). Design and characterization of synthetic fungal-bacterial consortia for direct production of isobutanol from cellulosic biomass. Proc Natl Acad Sci U S A.

[21] Ivanov I, Lo KC, Hawthorn L, Cowell JK, Ionov Y (2007). Identifying candidate colon cancer tumor suppressor genes using inhibition of nonsense-mediated mRNA decay in colon cancer cells. Oncogene.

[22] Li C, Lin F, Li Y, Wei W, Wang H, Qin L, Zhou Z, Li B, Wu F, Chen Z (2016). A beta-glucosidase hyper-production Trichoderma reesei mutant reveals a potential role of cel3D in cellulase production. Microb Cell Fact.

[23] Nasif S, Contu L, Muhlemann O (2018). Beyond quality control: the role of nonsense-mediated mRNA decay (NMD) in regulating gene expression. Semin Cell Dev Biol.

[24] Chamieh H, Ballut L, Bonneau F, Le Hir H (2008). NMD factors UPF2 and UPF3 bridge UPF1 to the exon junction complex and stimulate its RNA helicase activity. Nat Struct Mol Biol.

[25] Xie BB, Li D, Shi WL, Qin QL, Wang XW, Rong JC, Sun CY, Huang F, Zhang XY, Dong XW (2015). Deep RNA sequencing reveals a high frequency of alternative splicing events in the fungus Trichoderma longibrachiatum. BMC Genom.

[26] Kim E, Magen A, Ast G (2007). Different levels of alternative splicing among eukaryotes. Nucleic Acids Res.

[27] Wang B, Guo G, Wang C, Lin Y, Wang X, Zhao M, Guo Y, He M, Zhang Y, Pan L (2010). Survey of the transcriptome of Aspergillus oryzae via massively parallel mRNA sequencing. Nucl Acids Res.

[28] Marquez Y, Brown JW, Simpson C, Barta A, Kalyna M (2012). Transcriptome survey reveals increased complexity of the alternative splicing landscape in Arabidopsis. Genome Res.

[29] Braunschweig U, Barbosa-Morais NL, Pan Q, Nachman EN, Alipanahi B, Gonatopoulos-Pournatzis T, Frey B, Irimia M, Blencowe BJ (2014). Widespread intron retention in mammals functionally tunes transcriptomes. Genome Res.

[30] Gonzalez-Hilarion S, Paulet D, Lee KT, Hon CC, Lechat P, Mogensen E, Moyrand F, Proux C, Barboux R, Bussotti G (2016). Intron retention-dependent gene regulation in Cryptococcus neoformans. Sci Rep.

[31] Kumar S, Mutturi S (2021). Alternative splicing regulates the alpha-glucosidase synthesis in Aspergillus neoniger NCIM 1400. Fungal Biol.

[32] Goebels C, Thonn A, Gonzalez-Hilarion S, Rolland O, Moyrand F, Beilharz TH, Janbon G (2013). Introns regulate gene expression in Cryptococcus neoformans in a Pab2p dependent pathway. PLoS Genet.

[33] Yap K, Lim ZQ, Khandelia P, Friedman B, Makeyev EV (2012). Coordinated regulation of neuronal mRNA steady-state levels through developmentally controlled intron retention. Genes Dev.

[34] Moran Y, Weinberger H, Reitzel AM, Sullivan JC, Kahn R, Gordon D, Finnerty JR, Gurevitz M (2008). Intron retention as a posttranscriptional regulatory mechanism of neurotoxin expression at early life stages of the starlet anemone Nematostella vectensis. J Mol Biol.

[35] Middleton R, Gao D, Thomas A, Singh B, Au A, Wong JJ, Bomane A, Cosson B, Eyras E, Rasko JE (2017). IRFinder: assessing the impact of intron retention on mammalian gene expression. Genome Biol.

[36] Lareau LF, Inada M, Green RE, Wengrod JC, Brenner SE (2007). Unproductive splicing of SR genes associated with highly conserved and ultraconserved DNA elements. Nature.

[37] Wong JJ, Ritchie W, Ebner OA, Selbach M, Wong JW, Huang Y, Gao D, Pinello N, Gonzalez M, Baidya K (2013). Orchestrated intron retention regulates normal granulocyte differentiation. Cell.

[38] Filichkin SA, Mockler TC (2012). Unproductive alternative splicing and nonsense mRNAs: a widespread phenomenon among plant circadian clock genes. Biol Direct.

[39] Pan Q, Saltzman AL, Kim YK, Misquitta C, Shai O, Maquat LE, Frey BJ, Blencowe BJ (2006). Quantitative microarray profiling provides evidence against widespread coupling of alternative splicing with nonsense-mediated mRNA decay to control gene expression. Genes Dev.

[40] Weischenfeldt J, Waage J, Tian G, Zhao J, Damgaard I, Jakobsen JS, Kristiansen K, Krogh A, Wang J, Porse BT (2012). Mammalian tissues defective in nonsense-mediated mRNA decay display highly aberrant splicing patterns. Genome Biol.

[41] Kim J, Guan KL (2019). mTOR as a central hub of nutrient signalling and cell growth. Nat Cell Biol.

[42] Heitman J, Movva NR, Hall MN (1991). Targets for cell cycle arrest by the immunosuppressant rapamycin in yeast. Science.

[43] Morgan JT, Fink GR, Bartel DPJN (2019). Excised linear introns regulate growth in yeast. Nature.

