# Supplementary material for: Intron retention coupled with nonsense-mediated decay is involved in cellulase biosynthesis in cellulolytic fungi
Source: Biotechnol Biofuels Bioprod. 2022 May 19;15:53. doi: 10.1186/s13068-022-02141-x (PMC9118705; doi:10.1186/s13068-022-02141-x)
Supplement: Supplementary file 1 — Additional file 1: Table S1. Primers used for RT-qPCR in this study. Fig. S1. Relative levels of introns in cel7a, cel7b, and cel3a in T. reesei RUT-C30 cultivated for 7 days in TMM containing 2% cellulose, 2% lactose or 2% glucose. Data are represented as the mean of three independent experiments and error bars express the standard. [file 13068_2022_2141_MOESM1_ESM.docx]

**Intron retention coupled with nonsense-mediated decay is involved in cellulase biosynthesis in cellulolytic fungi**

**Yichen Gao,^1^ Ai-Ping Pang,^1^ Leyao Ma,^1^ Haiyan Wang,^1^ Samran Durrani,^1^ Bingzhi Li,^2^ Fu-Gen Wu,^1^ Fengming Lin^1*^**

^1^ State Key Laboratory of Bioelectronics, School of Biological Science and Medical Engineering, Southeast University, Nanjing, China

^2^ Key Laboratory of Systems Bioengineering (Ministry of Education), School of Chemical Engineering and Technology, Tianjin University, Tianjin, China

*Correspondence: [linfengming@seu.edu.cn](mailto:linfengming@seu.edu.cn)

**Table S1** The primers used for RT-qPCR in this study.

| Primer | Sequence (5’ – 3’) |
| --- | --- |
| cel7a-i-F | GACGCTATCTTCTTGTTGGC |
| cel7a-i-R | CACTGGCTGTCACAGTACCC |
| cel7a-ii-F | TACTGCACAGCTGAGGAGGC |
| cel7a-ii-R | ACTCTTTGTCAACGCGCATG |
| cel7a-F | CGACTGTCGGCCAGGAGATC |
| cel7a-R | GTATGGGTTCCAGTCGCAGC |
| cel7b-i-F | GTGAGCCTGATGCCACTACT |
| cel7b-i-R | GTTGAACTGGGTGATGATGG |
| cel7b-ii-F | CAGTGCGGTGGCATTGGGTA |
| cel7b-ii-R | ATCACTCCCGTCAGGCATGG |
| cel7b-F | CTGGAACTACCGCTGGATGC |
| cel7b-R | GACGCCCTCGATGAAGCAGT |
| cel3a-i-F | GTATCCTGGAGACACCATGC |
| cel3a-i-R | GAGATTGAGCTTTGCCAATG |
| cel3a-ii-F | ACTATAAGCACTTCGACGAC |
| cel3a-ii-R | CTATCATTCCACAGTCATCC |
| cel3a-F | GGATCAGTACACGCTGCAGA |
| cel3a-R  sar1-F  sar1-R | CCGATTGTTACCGTTGAAGT  TGGATCGTCAACTGGTTCTACGA  GCATGTGTAGCAACGTGGTCTTT |


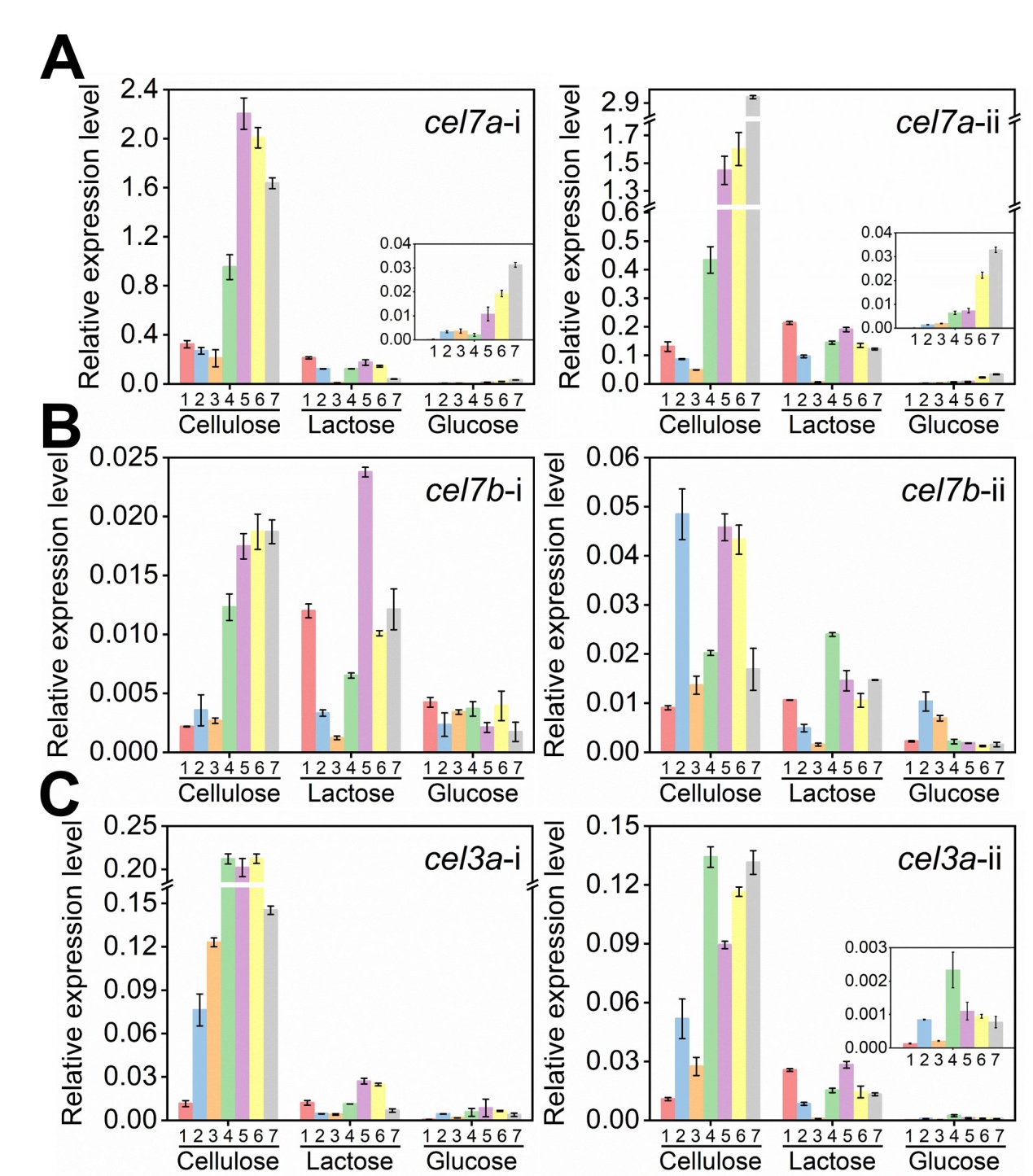


**Fig. S1** The relative levels of introns in *cel7a*, *cel7b*, and *cel3a* in *T*. *reesei* RUT-C30 cultivated for 7 days in TMM containing 2% cellulose, 2% lactose or 2% glucose. Data are represented as the mean of three independent experiments and error bars express the standard.
